# In search of a gold standard patient-reported outcome measure to use in the evaluation and treatment-decision making in migraine prevention. A real-world evidence study

**DOI:** 10.1186/s10194-021-01366-9

**Published:** 2021-12-13

**Authors:** Alicia Alpuente, Victor J Gallardo, Edoardo Caronna, Marta Torres-Ferrus, Patricia Pozo-Rosich

**Affiliations:** 1 Headache Unit, Neurology Department, Vall d’Hebron University Hospital, Barcelona, Spain; 2 Headache and Neurological Pain Research Group, Vall d’Hebron Research Institute, Departament de Medicina, Universitat Autònoma de Barcelona, Barcelona, Spain; 3 Headache Unit, Neurology Department, Hospital Universitari Vall d’Hebron, 119-129 Passeig de la Vall d’Hebron, 08035 Barcelona, Spain

**Keywords:** Headache, Migraine, Outcomes, Patient-reported outcome, Health-related quality of life, Efficacy, anti-CGRP monoclonal antibodies

## Abstract

**Background:**

Patient-Reported Outcomes (PROs) have been developed to numerically quantify disability, impact and quality of life. They have been widely used in migraine clinical trials. However, we still do not know which PRO more accurately reflects preventive treatment response from a patient’s perspective or which one may help us with treatment decisions in clinical practice. They have been used to enforce the efficacy results in clinical trials and real-world evidence so far. The aim of this study was to analyze which PROM is (1) better correlated with all primary efficacy endpoints and (2) which one is better associated with treatment continuation with CGRP-mAbs at week-12, which is usually the moment when this decision is made.

**Methods:**

Patients with migraine who had received 3 administrations of CGRP-mAbs were evaluated in this prospective cohort study. Primary efficacy outcomes considered: a change in migraine days (MMD), headache days (MHD), pain intensity (INT), acute medication days (AMD) and 50% responder rate. The Spearman coefficient (r_s_) was the measure used for quantify the strength of the correlation between PROMs and treatment efficacy outcomes changes. A stepwise logistic regression identified which PROM was independently associated with treatment continuation at week-12.

**Results:**

263 patients completed 12 weeks of treatment. The efficacy outcomes and PROMs scores were statistically significantly reduced at week-12 for all patients. The role function-restrictive (RFR) domain of the Migraine-Specific Quality of Life (MSQ) questionnaire was statistically significantly correlated with all primary efficacy outcomes. Relative changes in MSQ total score (OR[95%]: 0.840[0.619-0.973]; *p*=0.037) and Patient Global Impression of Change (PGIC) scale (OR[95%]: 15.569[6.254-31.533]; *p*<0.001) were the PROMs associated with treatment continuation as independent factors at week-12.

**Conclusions:**

Changes in MSQ questionnaire and PGIC scale at week-12 were the PROMs with higher association with CGRP-mAbs response from a patient’s perspective and medical decision-taking.

**Supplementary Information:**

The online version contains supplementary material available at 10.1186/s10194-021-01366-9.

## Background

Migraine is a chronic neurological disease consisting on a predisposition to suffer from disabling episodic attacks of headache and accompanying symptoms [1]. It affects over 1 billion people worldwide with an impact on individuals, families, work and society [2, 3]. According to the Global Burden of Disease Study 2016, migraine is the second leading cause of disability overall, and the leading cause of disability in people younger than 50 years (particularly in women) [4]. As a result, the health-related quality of life (HRQoL) is reduced in migraine patients.

According to the International Headache Society (IHS) guidelines, preventive treatments are intended to reduce frequency, intensity of headache pain and use of acute medication [5]. Therefore, reduction in monthly migraine and headache days has been considered the primary efficacy endpoints per excellence in randomized controlled trials of migraine preventive treatments [6]. Other efficacy endpoints used are the response rate of patients who decrease headache/migraine frequency (50, 75 or 100%, reduction in acute medication intake and improvement in severity of migraine attacks. Over the years, however, these endpoints, which are objective and quantitative, have been complemented with more patient-centered, subjective and personal outcome measures. These measures are the Patient-Reported Outcomes (PROMs), defined as self-reported measures on symptoms, functional status and perceptions [7]. They are important tools for capturing information from the patient’s perspective and should be used to evaluate impact on the HRQoL, functional and emotional disability, and specifically in migraine, to evaluate the evolution of patients after initiating preventive treatment.

The arrival of the new therapies targeting calcitonin gene related peptide (CGRP) has started a new era in migraine [8]. In particular, CGRP-mAbs have been the first target-driven treatment to be approved for migraine prevention. Their efficacy and safety have been demonstrated in randomized controlled trials (RCT) as well as, in real-world evidence (RWE) studies [9–13]. Both RCT and post-approval RWE studies have included PROMs and healthcare outcomes in order to enforce the efficacy results [14]. However, despite the large number of available PROMs, most lack clarity with regard to measurement aim and have limited evidence of essential measurement properties, limiting confidence in data interpretation [15].

In clinical practice with CGRP-mAbs the evaluation and decision on treatment continuation at week-12 is extremely important. This is especially relevant when CGRP-mAbs are prescribed not only because they have a quicker efficacy response than oral prophylactics or OnabotulinumtoxinA, but also because local regulatory agencies quarterly demand clinicians a rationale in their use. It is also important to point out that clinicians have an important responsibility in deciding the efficacy evaluation and treatment continuation, and valuable complementary information would be used in clinical practice. Clinicians who are familiar in prescribing CGRP-mAbs know that there are several cases in which this assessment is not so clear. There are patients who cannot clearly be categorized as non- or excellent- responders. It is in these cases when an effort has to be made and integrate both objective and subjective outcomes measures, for the benefit of the patient; taking into account, that there is no consensus of which PRO measure better captures treatment efficacy from a patient’s perspective [16, 17]. In addition, time is limited in clinical routine practice and selecting the best PROMs is critical to optimize the migraine standard of care considering the following three mainstays: (1) more accurately correlated with IHS guideline treatment endpoints [6], (2) more reliable for medical decision-making on treatment continuation and (3), stronger evidence that improvement reflects patient’s perspective.

Hence, the aim of this study was to analyze which PROM was (1) better correlated with all primary efficacy endpoints and (2) which one was better associated with treatment continuation with CGRP-mAbs at week-12, which is usually the moment when this decision is made.

## Methods

### Study population

This was a prospective cohort study conducted at the outpatient Headache Clinic based in a public third-level University Hospital. Consecutive migraine patients who were candidates to start preventive treatment with CGRP-mAbs according to our national regulatory agency were included [18] during two years (since February 2019 to February 2021). Migraine was diagnosed by a neurologist according to the International Headache Society criteria 3rd edition (ICHD-3) [19]. mAbs were initiated according to national requirements: more than 8 migraine days per months and previous history of treatment failure to 3 preventive treatments, being OnabotulinumtoxinA one of them in chronic migraine patients.

### Clinical variables

Clinical variables were collected by an electronic diary completed daily by participants. Primary efficacy endpoints considered in this study where: a change in monthly migraine days (MMD), a change in monthly headache days (MHD), a change in headache pain intensity (INT, scored from 0 to 3, 0 being without pain and 3 severe pain), a change in acute medication intake days (AMD), a change in the acute medication burden (defined as the number of pills/month or AMPM) and the 50% responder rate. We included the most widely-used PROMs in our clinical practice: Patient Global Impression of Change (PGIC) scale [20], Migraine-Specific Quality of Life (MSQ) questionnaire v2.1 [21], Headache Impact Test (HIT-6) score [22] and Migraine Disability Assessment (MIDAS) questionnaire [23]. We also evaluated co-morbid psychiatric changes using validated questionnaires: the Beck Depression Inventory (BDI-II) [24], the Beck Anxiety Inventory (BAI) [25] and the MIGraine attacks - Subjective COGnitive impairments scale (MIG-SCOG) [26]. PROMs and the other questionnaires were completed at baseline and after 3 treatment administrations (week-0 and week-12). All patients completed all the questionnaires regardless of their treatment response.

### Preventive treatment

CGRP-mAbs were administered monthly (Erenumab 140 mg or Galcanezumab 120 mg with a loading dose of 240 mg). Treatment response was evaluated after 3 administrations at week-12. A primary analysis of our initial cohort was previously published [27]. Patients were divided into two groups depending on whether they continued treatment after 12-weeks (GO group) or stopped it (no-GO group). Unacceptable treatment tolerability, not achieving at least a 30% reduction in MHD and/or 30% improvement in pain intensity were criteria for stopping treatment at week-12.

### Statistical analysis

We reported nominal (categorical) variables as frequencies (percentages) while continuous variables as mean ± standard deviation when normally distributed or median and interquartile range (IQR) when non-normally distributed. We checked normality assumption of quantitative variables through visual methods (Q-Q plots) and statistical test (Shapiro-Wilk test). We assessed statistical significance between baseline (week-0) and after 3-months (week-12) using paired t-test for continuous variables with normal distribution and paired Mann-Whitney U test for the rest. Pearson’s chi-square was used when comparing categorical variables between treatment timepoints. We assessed statistical significance between the GO and No-GO group using the same statistical tests under equal assumptions previously described but, within independent groups.

In order to study the correlation between PROMs/questionnaires and treatment efficacy outcomes improvement, relative change (%∆) was computed for all variables (week-0 and week-12). Then, Spearman’s rank-order correlation (r_s_) was calculated as a measure of strength and direction of pairwise contrast on relative change between quantitative measurements. In the case of PGIC, we evaluated its association with %∆ in clinical variables through Kruskal-Wallis test with Bonferroni adjustment in pairwise comparisons (improvement, no-change and worsening).

For our preplanned multivariate analysis, both (forward and backward) stepwise logistic regression was used in order to identify which PRO/questionnaire were independently associated with treatment continuation at week-12. Included variables were either based on statistical significance from bivariate tests (p≤0.10) or considered clinically relevant (disease evolution time and mAb treatment). The final set of features (predictors) was chosen according to their effect in minimizing (optimizing) the Akaike Information Criteria (AIC) of the resulting model. Classification model was computed in the 75% of initial sample (randomly selected as a train subset) and tested in the rest of data (25% as a test subset). A 10-fold cross-validation (10-fold CV) was also performed in order to validate the prediction outcomes of our logistic regression classifier, assessing the model performance by their accuracy, kappa value and the area under the curve (AUC) in both subsets (train and test). Multicollinearity between potential predictors was assessed with the calculation of variance inflation factors (VIF).

Finally, a receiver operating characteristic curve (ROC) was performed to identify the most clinically valid cut-off point for the relative change (%∆) of the independent PROs/questionnaires scores identified from the multivariate analysis in relation to the continuation of treatment. Cut-off selection from ROC was computed using Youden’s index.

We did not conduct a statistical power calculation prior to the study because the sample size was based on the available data. No missing values were obtained. No adjustment for multiple comparisons was made to the statistical inferences, but exact P values were reported to allow post adjustments. We considered two-tailed test P values <0.05 as statistically significant. All analyses were done using R Core Team, 2021 (v4.0.5).

### Ethics approval and patients’ consent

The study was approved by the Vall d’Hebron Ethics Committee (PR(AG)53/2017). All patients gave an informed consent for the analysis of patients’ data. All patients consented to publication of anonymous individual data.

## Results

### Treatment response

Since February 2019, 359 patients started treatment with mAbs. At the time of this analysis, 73.3% (263/359) had completed the 12 weeks of treatment (62.4% Erenumab and 37.6% Galcanezumab). In this cohort, 81.7% were females. The mean age was 46.9±10.3 years old. Regarding diagnosis, 87.8% (231/263) fulfilled criteria for chronic migraine (CM) and 68.4% (180/263) for medication overuse (MO). Demographic and clinical data are summarized in Table 1.

**Table 1:** Baseline characteristics and basal differences between patients who continue with CGRP-mAb treatment (GO) and patients who discontinue (No-GO) after 3-months

	Total (*n* = 263)	GO (*n*=219)	No-GO (*n*=44)	*P-value*
**Demographics**				
Age, mean (SD) years	46.9 (10.3)	46.9 (10.4)	46.8 (10.1)	0.981^†^
Gender (female), n (%)	215 (81.7)	181 (82.6%)	34 (77.3%)	0.399^§^
**Disease characteristics**				
Diagnosis, n (%)				
EM CM	32 (12.2) 231 (87.8)	30 (93.8%) 189 (81.8%)	2 (6.2%) 42 (18.2%)	0.056^§^
Duration of migraine disease, mean (SD) years	25.4 (13.5)	25.9 (13.2)	22.8 (14.6)	0.118^‡^
Chronification time^*^, mean (SD) years	9.7 (8.5)	9.8 (8.6)	9.3 (8.3)	0.956^‡^
Aura, n (%)	75 (28.5)	60 (27.4%)	15 (34.1%)	0.364^§^
MHD, mean (SD), d/mo MMD, mean (SD), d/mo Headache pain intensity, mean (SD), 0-3 score	22.2 (6.8) 16.7 (7.0) 1.59 (0.56)	21.5 (6.7) 15.9 (6.9) 1.53 (0.55)	25.4 (5.8) 20.5 (6.4) 1.90 (0.50)	<0.001^‡^ <0.001^‡^ <0.001^†^
**Preventive treatment**				
CGRP-mAb Treatment, n (%)				
Erenumab 140 mg Galcanezumab 120 mg	164 (62.4) 99 (37.6)	143 (87.2%) 76 (76.8%)	21 (12.8%) 23 (23.2%)	0.072^§^
Concomitant preventive treatment, n (%)	176 (66.9%)	136 (62.1%)	40 (90.9%)	<0.001^§^
Prior preventive classes failures, median [IQR]	4.0 [1.0]	4.0 [1.0]	4.0 [1.2]	0.448^‡^
**Acute medication**				
Acute medication frequency, mean (SD), d/mo	15.5 (8.6)	14.8 (8.1)	19.1 (9.8)	0.008^‡^
Acute medication burden, mean (SD), p/mo	21.2 (13.3)	20.7 (13.0)	24.0 (14.3)	<0.001^‡^
Medication overuse, n (%)	180 (68.4)	148 (67.6%)	32 (72.7%)	0.486^§^
**Migraine-related clinical burden (PROMs)**				
Disability (MIDAS), median [IQR]	59.0 [81.5]	57.0 [80.0]	78.5 [152.5]	0.008^‡^
Headache-related impact (HIT-6), mean (SD)	67.7 (24.0)	67.9 (25.8)	67.0 (11.1)	0.078^‡^
Quality of life (MSQ_T_), mean (SD)	32.8 (17.2)	34.1 (16.7)	26.4 (18.4)	0.004^‡^
Anxiety (BAI), median [IQR]	19.0 [22.0]	18.0 [20.0]	29.5 [24.0]	0.001^‡^
Depression (BDI-II), median [IQR]	13.0 [17.0]	12.0 [17.0]	18.2 [15.2]	0.004^‡^
Cognitive impairment (MigScog), mean (SD)	9.3 (5.0)	9.2 (5.1)	10.0 (4.7)	0.230^‡^

^*^Data available only from CM patients (231/268)

^**§**^Significance assessed with Fisher’s exact test or linear trend chi-square test (preventive classes failures)

^**†**^Significance assessed with independent *t*-test

^**‡**^Significance assessed with Mann-Whitney U test

Abbreviations: SD: standard deviation; IQR: interquartile range; d/mo: days per month; p/mo: pills per month; CM: chronic migraine; EM: episodic migraine; MHD: monthly headache days; MMD: monthly migraine days; MIDAS: migraine disability assessment; HIT-6: headache impact test; MSQ_T_: migraine-specific quality of life questionnaire (total score); BAI: Beck anxiety inventory; BDI-II: Beck depression inventory-second edition; MIG-SCOG: migraine attacks-subjective cognitive impairment scale

At week-12, 83.3% (219/263) of patients continued treatment and 16.7% (44/263) stopped it. Three patients (6.8%, 3/44) stopped treatment because of both a lack of tolerability and efficacy. The mean change (SD) in MMD was -7.9±7.4 days/month (week-0: 16.7±7.0 vs. week-12: 8.8±7.4; *p*<0.0001). The rest of the treatment efficacy outcomes were statistically significantly reduced after 12 weeks of treatment (see Supp. Fig. 1A). Moreover, 54.4% (143/263) had a 50% or greater reduction (response rate, RR) in MMD while 41.1% (108/263 of patients) in MHD. PROMs and questionnaires scores were also statistically significantly reduced after 12 weeks of treatment (see Supp. Fig. 1 B). Neither statistically significant differences were found in ≥50% RR between mAbs in MMD (Erenumab: 57.9% vs. Galcanezumab: 48.5%; *p*=0.160) nor MHD (Erenumab: 44.5% vs. Galcanezumab: 35.4%; *p*=0.156). Hence, we assumed a comparable improvement for both treatments.

Patients who continued treatment at week-12 had a 50% or greater reduction in in MMD (GO: 62.6% vs. No-GO: 13.6%; *p*<0.0001) and MHD (GO: 48.4% vs. No-GO: 4.5%; *p*<0.0001). We also observed that relative change (%∆) in all the treatment efficacy outcomes (see Supp. Fig. 2A) and all PROMs/questionnaires scores (see Supp. Fig. 2B), were statistically significantly reduced in the GO group, with the exception of the MIG-SCOG scale.

When we compared GO and No-GO group, we found that patients in the latter group significantly presented higher headache frequency, intensity and acute treatment intake at baseline (Table 1). This group also showed significantly higher score in MIDAS, MSQ, BAI and BDI-II questionnaires but lower score in the MSQ total score (MSQ_T_). We also found that concomitant preventive treatment was statistically significantly associated with No-GO group (Table 1). However, no statistically significant differences were found between patients’ discontinuation and type of mAb (Erenumab: 12.8% vs. Galcanezumab: 23.2%; *p*=0.072).

### PROMs evaluation

**Fig. 1:**
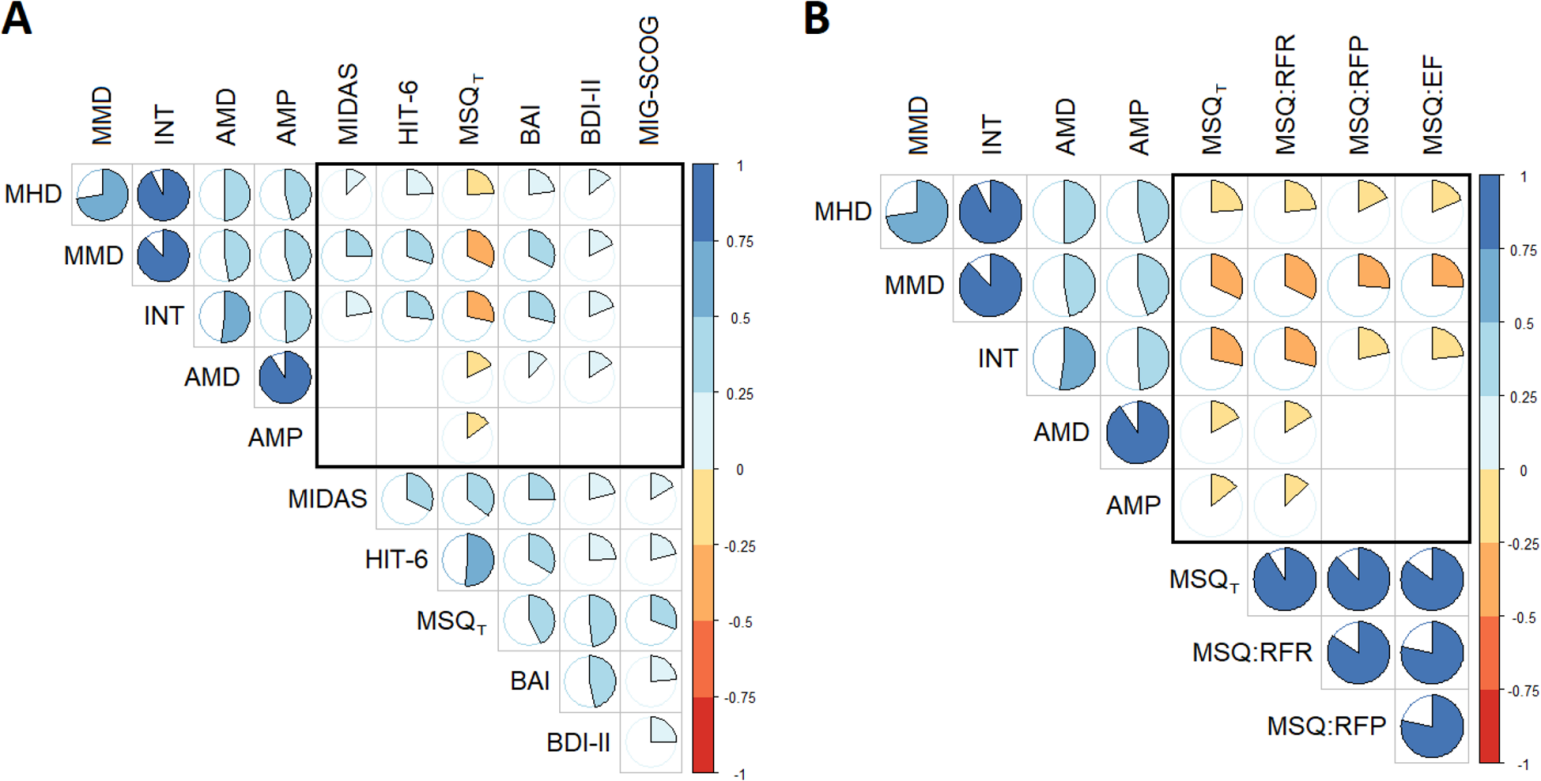
Relative changes (∆%) correlations between treatment efficacy outcomes improvement and PROMs changes (**A**: MIDAS, HIT-6, MSQ_T_, BAI, BDI-II, MIG-SCOG; **B**: MSQ_T_, MSQ_RFR_, MSQ_RFP_, MSQ_EF_). Pie charts represent the strength of the correlation (r_s_) between pairwise ∆ and color the direction of this relationship (blue: positive, red: negative). Blank cells refer to non-statistically significantly correlation between pairwise ∆. Abbreviations MHD: monthly headache days; MMD: monthly migraine days; INT: headache pain intensity; AMD: days of acute medication intake; AMP: acute medication burden (pills/month); MIDAS: migraine disability assessment; HIT-6: headache impact test; MSQ_T_: migraine-specific quality of life questionnaire (total score); MSQ-RFR: MSQ role-function restrictive; MSQ-RFP: MSQ role-function preventive; MSQ-EF: MSQ emotional function; BAI: Beck anxiety inventory; BDI-II: Beck depression inventory-second edition; MIG-SCOG: migraine attacks-subjective cognitive impairment scale.

In order to analyze which PROM or questionnaire was better correlated with an overall improvement in traditional treatment efficacy outcomes, we measured the significance correlation between the corresponding relative changes (∆%). Only the MSQ total score (MSQ_T_) was correlated with an improvement in all the treatment efficacy outcomes: MMD (r_s_=0.321; *p*<0.0001); MHD (r_s_=0.243; *p*<0.0001); INT (r_s_=0.285; *p*<0.0001); AMD (r_s_=0.172; *p*=0.004) and AMPM (r_s_=0.147; *p*=0.016) (see Fig. 1A). From the three domains of the MSQ, the role function-restrictive (RFR) domain was the only one with a statistically significant correlation with all the treatment efficacy outcomes (see Fig. 1B). MIG-SCOG was the only scale without any statistically significant correlation. Finally, we also observed that improvement in PGIC scale was statistically significantly associated with ∆% reduction in all the treatment efficacy outcomes (see Supp. Fig. 3).

### Treatment continuation evaluation

**Fig. 2:**
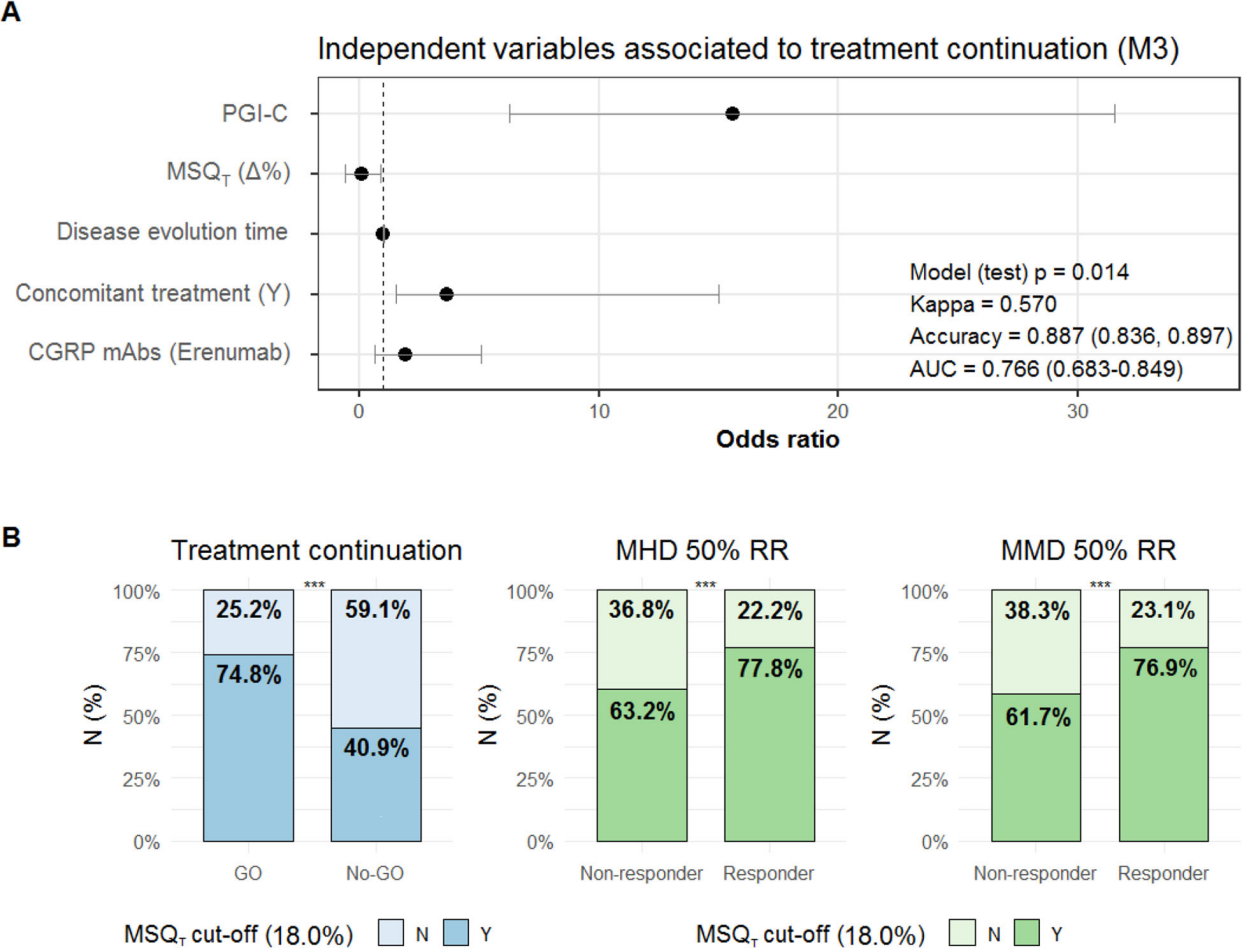
Odds Ratio (95% CI) estimated from the logistic regression analysis of the significant clinical predictors associated to CGRP-mAb treatment continuation (**A**) and statistical significance of the MSQ_T_ cut-off and treatment response (**B**). (**A**) Odds ratio (95% CI) estimated from the 10-fold CV of the stepwise logistic regression with selection criteria of minimizing the AIC for a variable to be eliminated from the selected model. (**B**) ^***^P-value < 0.0001; significance assessed with Fisher’s exact test. MSQ cut-off selection from ROC using Youden’s index. Abbreviations: PGI-C: patient global impression scale; MSQ_T_: migraine-specific quality of life questionnaire (total score); MHD: monthly headache days; MMD: monthly migraine days; RR.

After the AIC stepwise selection of the potential predictors for treatment continuation (basal diagnosis, preventive treatment, PGIC and ∆% from all PROMs/questionnaires) and the clinically relevant covariates (disease evolution time and type of mAb), the final predictors introduced in the logistic regression multivariate analysis were: concomitant preventive treatment (N/Y), disease evolution time, type of mAb (Erenumab/Galcanezumab), ∆% in MSQ_T_ and PGIC. We observed that concomitant preventive treatment (OR[95%]: 3.629[1.114-14.994]; *p*=0.037), ∆% in MSQ_T_ (OR[95%]: 0.840[0.619-0.973]; *p*=0.047) and PGIC (OR[95%]: 15.569[6.254-31.533]; *p*<0.001) remained as a statistically significant independent factors associated with treatment continuation (Fig. 2A). In the test set, the model presented an accuracy of 0.887 [0.836-0.897], a Kappa value of 0.570 (*p*=0.014) and AUC in the ROC of 0.766 [0.683-0.849].

The ROC analysis for the ∆% in the MSQ_T_ in relation to the decision on treatment continuation showed an AUC of 0.650 (95% CI, 0.610–0.795; *p*=0.013), suggesting that a mean increase of 18.0% in MSQ_T_ significantly predicted treatment continuation after 12 weeks of treatment (Fig. 2B). A statistically significantly association was also found with 18.0% MSQ_T_ increase and treatment response rates (Fig. 2B).

## Discussion

The goal of this RWE study was to quantify the relationship between primary treatment efficacy outcomes improvement and the change in PROMs commonly used in migraine in order to find and select the best one that summarizes the overall improvement (in terms of frequency, pain intensity and analgesic use). We found that an improvement in the MSQ and PGIC reflected a reduction in all treatment efficacy outcomes at week-12. Furthermore, a certain change in these scales accurately determined the continuation of treatment. Interestingly, changes in the MSQ score could predict the continuation of treatment beyond first term.

Migraine causes great impairment on the individual’s ability to participate in daily activities [28]. Hence, instruments that measure HRQoL are essential as they help us reflect this impact. In our clinical practice we use several instruments to assess treatment response in addition to the traditional efficacy outcomes, finding that, an improvement in daily-life function, measured by MSQ score, was correlated with a reduction in all the treatment efficacy outcomes (the RFR domain specifically). Furthermore, our study results indicated that a change in the MSQ score was an independent factor associated with treatment continuation. In particular, an 18% increase in MSQ significantly predicted treatment continuation at week-12 and it was associated to higher treatment response rates. In this regard, post-hoc analyses of the Galcanezumab clinical trials also found that MSQ scores increased with each successive level of migraine headache day response, and the greatest observed change was also for the RFR domain among the subgroup of patients experiencing ≥75% response rates [29].

The MSQ v2.1 is a self-administered 14-item instrument measuring 3 dimensions: role function–restrictive (RFR; 7 items assessing how migraines limit daily social and work-related activities), role function–preventive (RFP; 4 items assessing how migraine prevents doing these activities), and emotional function (EF; 3 items assessing the emotions associated with migraines) [30]. It is a reliable and validated instrument to measure HRQoL in patients with migraine, and can differentiate the impact of migraine across the headache-frequency spectrum (EM and CM) [31]. MSQ also helps monitor treatment response in migraine patients with preventive treatment [15]. Our findings show how important the ictal burden is, reflected by the restrictive domain, not only in clinical trials but also in clinical practice, due to limitations in daily activities such as work, household, childcare duties, and time with friends and family; and support the use of this instrument for assessing treatment response. MSQ is not usually used in clinical practice, in comparison to MIDAS and HIT-6; however, these results might guide us to use MSQ as a way of complementing the information provided by the headache diary.

We also found that the other PROM significantly associated with a reduction in all the treatment efficacy outcomes was the PGIC scale. This PROM is commonly used in mAbs clinical trials and it is correlated with significant treatment improvement [32, 33]. PGIC scale mainly measures change in clinical status. This PROMs is related to the patient’s perspective, which probably is the most important thing to take into account when evaluating the impact of treatment [20]. In fact, the patients’ perception of a treatment has been associated with treatment adherence [34]. So, asking our patients to rate the change in their migraine when taking a preventive treatment using the PGIC could be a valuable instrument since it is a very simple and short and probably should be introduced in clinical practice.

Traditional efficacy outcomes, as well as, some PROMs were developed and selected many years ago without migraine patient’s input. There is growing awareness about patient opinion and a suitable example is the outcome Most Bothersome Symptom (MBS). This outcome, which takes into account migraine attack symptoms that can disable equally or even more than headache, is increasingly being used in clinical trials for acute but also for preventive treatment in migraine [35, 36]. Other endpoints, such as total freedom of migraine, are not commonly used in clinical trials due to difficulties to achieve a statistical significance, but maybe, it is time to start using them in clinical practice.

It is important to stress that questionnaires measuring anxiety, depression and cognitive impairment were not correlated with an improvement in the treatment efficacy outcomes. Specifically, MIG-SCOG was the only questionnaire without any statistically significant correlation with treatment efficacy. They could be suitable instruments for assessing comorbidities or other dimensions of the disease rather than purely migraine. Moreover, these outcomes could improve in a long-term treatment. On the other hand, it is important to point out that HIT-6 and MIDAS scales’ were not significantly associated with either of these outcomes. The MIDAS scale is purely based on headache frequency whereas the HIT-6 scale is based on headache frequency and headache intensity. The MIDAS score is the sum of missed days due to a headache from paid work, housework, and non-work (family, social, leisure) activities and days where productivity was reduced by at least one-half [23]. The HIT-6 items measure the adverse impact of headache on social functioning, role functioning, vitality, cognitive functioning and psychological distress through frequency-related questions namely as *how often*. The HIT-6 also measures the severity of headache pain. So, they are reliable scales reflecting an improvement in migraine frequency but perhaps, they could not capture a global improvement in HRQoL.

A critical point in our decision-making in clinical practice is the information provided by guidelines. Nowadays, treatment guidelines consider improvements in patient functioning as an important outcome to be evaluated in order to decide the continuation of treatment [37, 38]. In this regard, The American Headache Society guidelines include specific guidance for MIDAS and HIT-6; however, they did not consider MSQ or PGIC. Our results showed that an increase of 18% in the MSQ score indicates treatment efficacy and therefore this information is a meaningful threshold to decide treatment continuation. A study including all of the pooled-data from the Galcanezumab clinical trials also analyzed meaningful change thresholds for the three domains of the MSQ at 3-months of treatment [39]. They found that the 3-month meaningful within-patient change thresholds were the same for EM and CM for RFP: 20.00 and EF: 26.67; and for RFR: 25.71. They also demonstrated that all three domains of the MSQ had sufficient reliability, validity, responsiveness, and appropriate interpretation standards. This is especially relevant when the assessment of the response based on classic endpoints is not clear enough, or CGRP-mAbs are prescribed by clinicians with less experience in this field.

According to our results, practical considerations arise for clinical management. This study could help us with two crucial questions in clinical practice: (1) treatment response assessment and (2) treatment continuation. Physicians are encouraged to use several treatment efficacy outcomes in order to assess treatment response, in particular with CGRP-mAbs since their clinical use is currently limited due to their proportional high cost and presence of Health Regulatory Authorities restrictions, which require a documented efficacy. Hence, a good option could be to use the PROM scale that better reflects the improvement in the rest of treatment-outcome measures, such as MSQ and PGIC, which will also help clinicians decide on whether to continue treatment or not and reflect the subjective impression of the patient. Our findings could help simplify management using reliable tools that translate an improvement in our patient’s quality of life.

Beyond these practical questions, this study should encourage reflection on the outcomes measures commonly used in clinical trials and thereby, in clinical practice, raising awareness about developing PROMs that better reflect patient priorities such as MBS, disability and stigma. Now that migraine treatment is dramatically changing, it is time to make an effort to ask patients and be able to transform patient’s thoughts in outcomes.

This study has some limitations. The lack of a control group makes it impossible to quantify the placebo effect; however, the efficacy results in our cohort are similar to other clinical trial and real-world series [40]. Data comes from a single headache center and is focused on CGRP-mAbs treatment response and PROMs used in this center. In future studies, we will have to determine if this is applicable to other migraine preventive treatments and other timepoints of treatment. Results shown are based on statistical significance, trying to find which PROMs is better associated to treatment improvement in migraine patients and clinical significance analysis should also be done in this field in order to properly study the degree of reproducibility of the current study findings. However, up to our knowledge, we report the first study to describe the global improvement of a preventive medication derived from a moderate-strength correlation with the relative change in PROMs. Finally, patients with CM in our study have an average 10 years of chronification, so, they are the most complex and difficult-to-treat patients.

This is the first study to evaluate in clinical practice the correlation between all of the objective and patient-centered subjective treatment efficacy outcomes, after 12 weeks of treatment with CGRP-mAbs. This timepoint is key when clinicians, together with patients, individually decide on whether to continue treatment or not. The MSQ and the PGIC scales can assist them in evaluating treatment efficacy by obtaining input directly from the patients on multidimensional aspects other than frequency of headache days or acute medication intake.

## Conclusions

In conclusion, MSQ (RFR) is the PROM which is significantly correlated with an improvement in all the treatment efficacy outcomes after 12 weeks of CGRP-mAbs. In addition, a change in the MSQ and PGIC scores are the most accurate way of predicting the continuation of CGRP-mAbs treatment at week-12. This is important as it gives clues on which PROMs better reflects a global treatment improvement, on which scale to use when evaluating treatment response and deciding on whether to continue treatment or not; and, probably, on which domain of migraine-burden do CGRP-mAbs treatment have a higher impact.

## Supplementary information

**Additional file 1.**

## Data Availability

Data are available upon request from the corresponding author.

## Abbreviations

HRQoL: Health-Related Quality Of Life
IHS: International Headache Society
PROMs: Patient-Reported Outcome Measurements
CGRP: calcitonin gene-related peptide
CGRP-mAbs: monoclonal antibodies targeting CGRP or its receptor
MHD: monthly headache days
MMD: monthly migraine days
INT: headache intensity
AMD: days of acute medication intake
AMB: acute medication burden
PGIC: Patient Global Impression of Change scale
MSQ: Migraine-Specific Quality of Life questionnaire
HIT-6: Headache Impact Test
MIDAS: Migraine Disability Assessment
BDI-II: Beck Depression Inventory
BDAI: Beck Anxiety Inventory
MIG-SCOG: MIGraine attacks-Subjective COGnitive impairments scale
VIF: variance inflation factors
ROC: receiver operating characteristic curve
RFP: role function–preventive
RFR: role function–restrictive
EF: emotional function
CM: chronic migraine
MO: medication overuse
RR: response rate
