# Supplementary material for: In search of a gold standard patient-reported outcome measure to use in the evaluation and treatment-decision making in migraine prevention. A real-world evidence study
Source: J Headache Pain. 2021 Dec 13;22(1):151. doi: 10.1186/s10194-021-01366-9 (PMC8903583; doi:10.1186/s10194-021-01366-9)
Supplement: Supplementary file 1 — Additional file 1. [file 10194_2021_1366_MOESM1_ESM.docx]

**Supplementary Figure 1.** Treatment efficacy outcomes (A) and PROMs scores reduction after 3-months of mAb treatment.


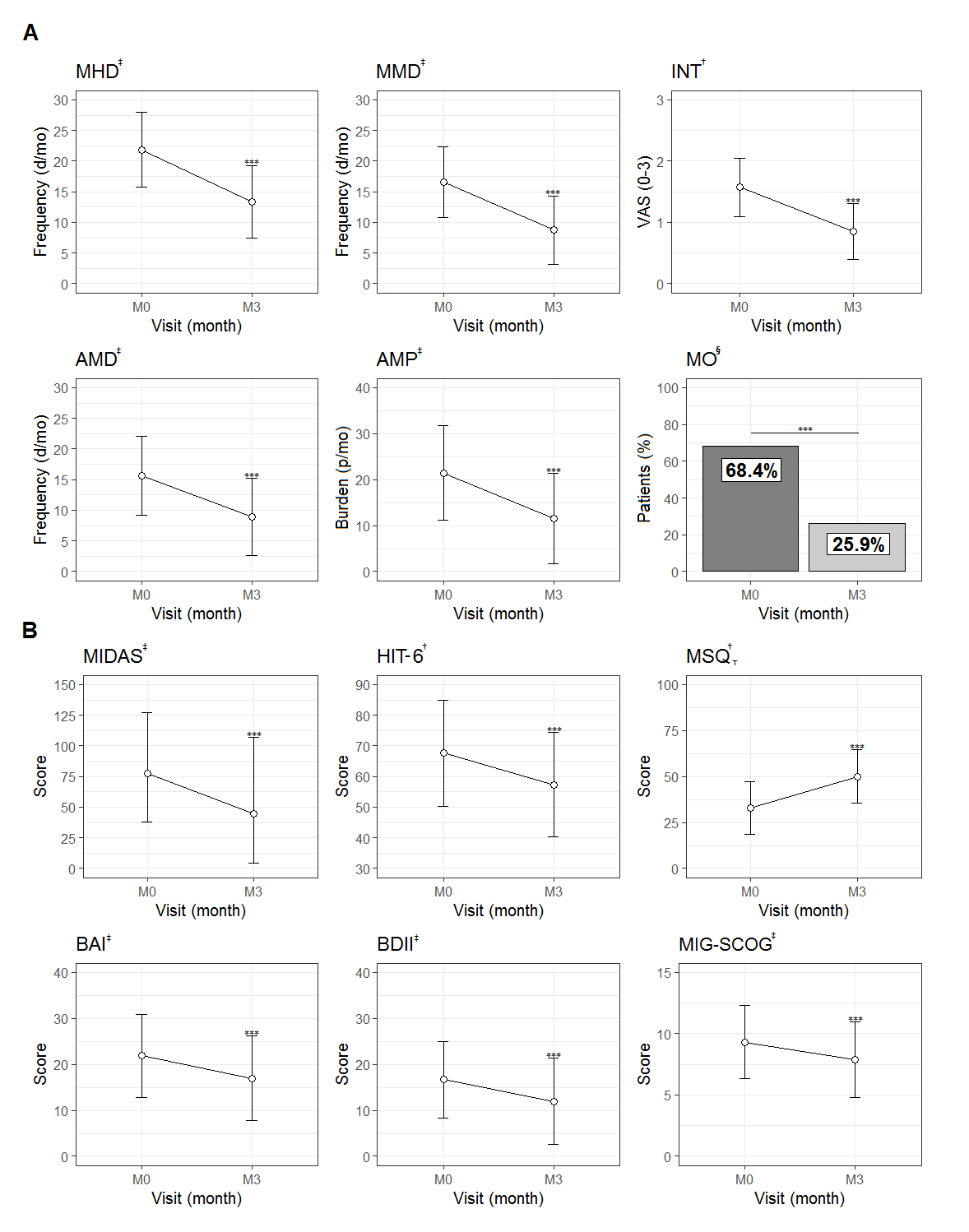


**^***^**P-value < 0.0001

**^§^**Significance assessed with Fisher’s exact test

**^†^**Significance assessed with paired *t*-test

**^‡^**Significance assessed with paired Mann-Whitney U test

Abbreviations: PROM: patient-reported outcome measure; d/mo: days per month; p/mo: pills per month; MHD: monthly headache days; MMD: monthly migraine days; INT: headache pain intensity; AMD: days of acute medication intake; AMP: acute medication burden or pills/month; MO: medication overuse; MIDAS: migraine disability assessment; HIT-6: headache impact test; MSQ_T_: migraine-specific quality of life questionnaire (total score); BAI: Beck anxiety inventory; BDI-II: Beck depression inventory-second edition; MIG-SCOG: migraine attacks-subjective cognitive impairment scale.

**Supplementary Figure 2.** Relative changes (%∆) from treatment efficacy outcomes (A) and PROMS questionnaires scores (B) after 3-months of mAb treatment between patients who continued treatment (GO) and patients who discontinued it (No-GO)


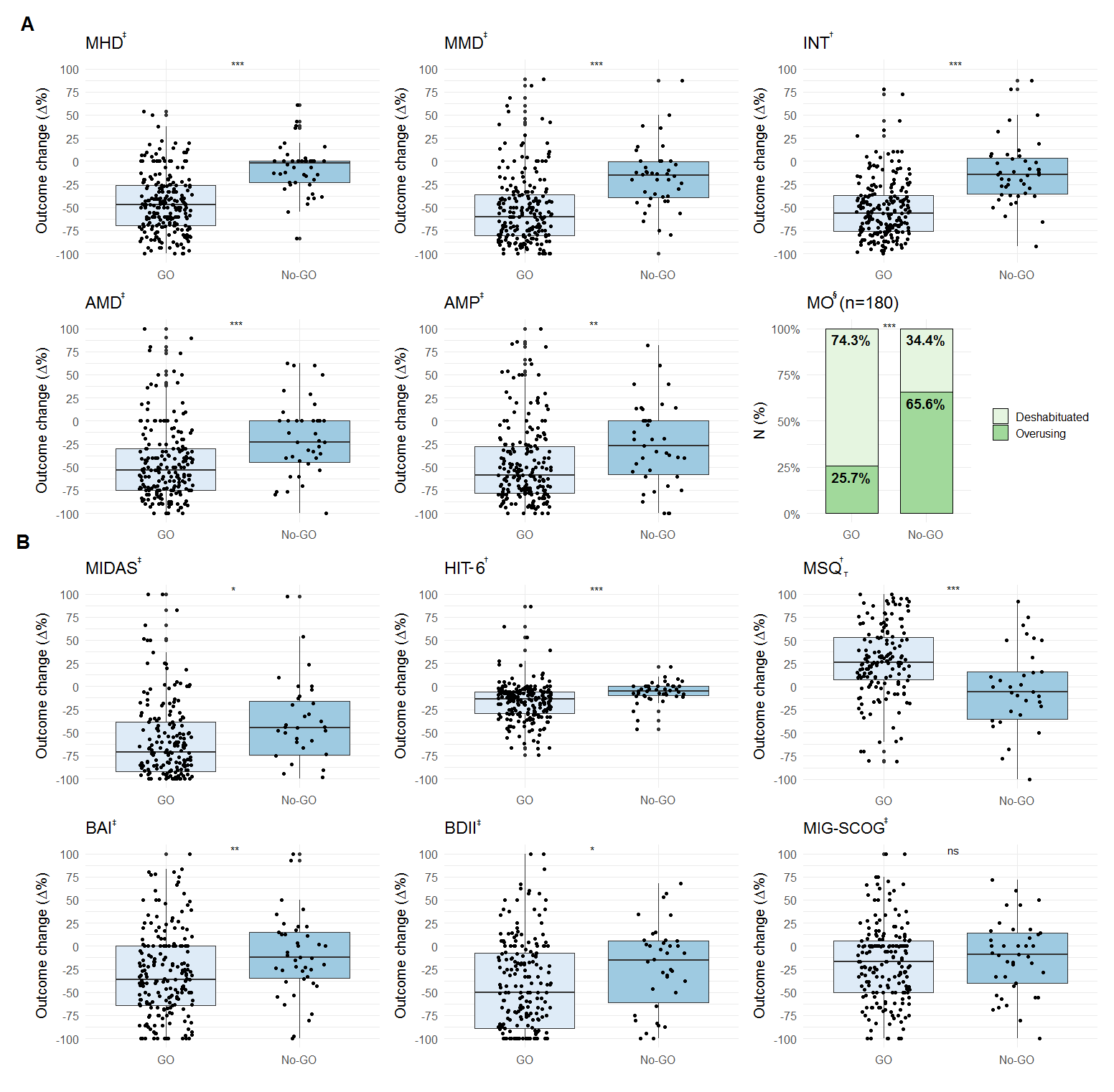


**^*^**P-value < 0.05; **^**^**P-value < 0.001; **^***^**P-value < 0.0001; ns: no statistically significant

**^§^**Significance assessed with Fisher’s exact test

**^†^**Significance assessed with independent *t*-test

**^‡^**Significance assessed with independent Mann-Whitney U test

Abbreviations: MHD: monthly headache days; MMD: monthly migraine days; INT: headache pain intensity; AMD: days of acute medication intake; AMP: acute medication burden or pills/month; MO: Medication Overuse; MIDAS: migraine disability assessment; HIT-6: headache impact test; MSQ_T_: migraine-specific quality of life questionnaire (total score); BAI: Beck anxiety inventory; BDI-II: Beck depression inventory-second edition; MIG-SCOG: migraine attacks-subjective cognitive impairment scale.

**Supplementary Figure 3.** Relative changes (∆%) associations between treatment efficacy outcomes improvement and PGI-C.


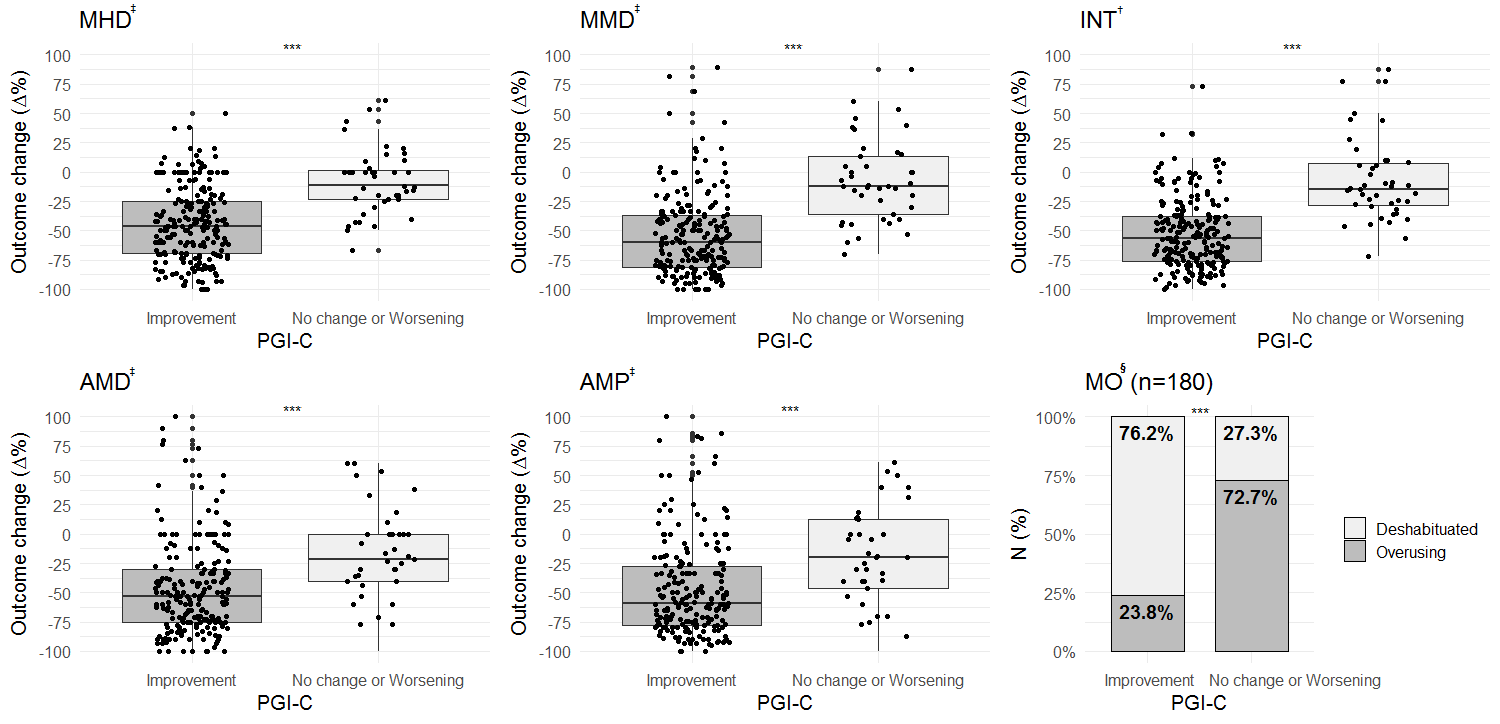


**^***^**P-value < 0.0001

**^§^**Significance assessed with Fisher’s exact test

**^†^**Significance assessed with independent *t*-test

**^‡^**Significance assessed with independent Mann-Whitney U test

Abbreviations: MHD: monthly headache days; MMD: monthly migraine days; INT: headache pain intensity; AMD: days of acute medication intake; AMP: acute medication burden or pills/month; MO: Medication Overuse; PGI-C: patient global impression scale
